# Corrigendum

**DOI:** 10.2471/BLT.15.100615

**Published:** 2015-06-01

In Volume 93, Issue 5, May 2015, page 360, Fig. 1 should be as follows.

**Fig. 1:**
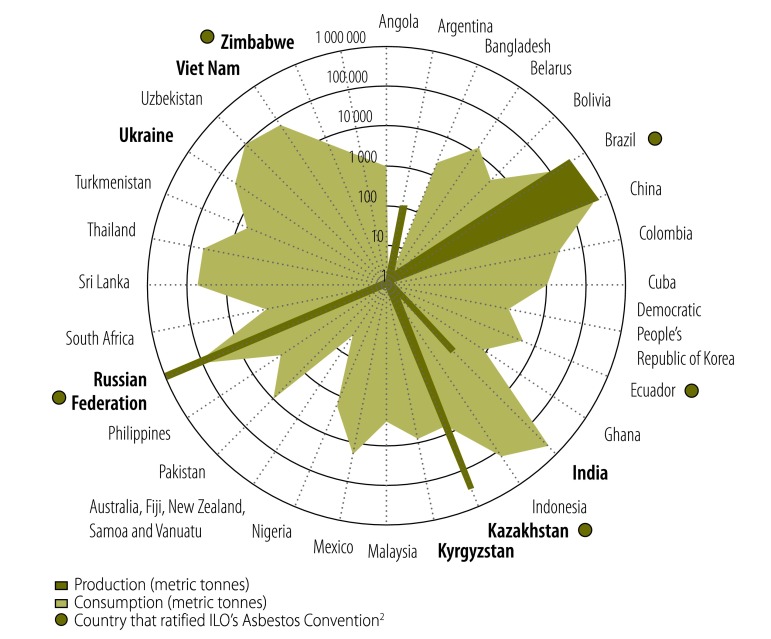
Asbestos producers and consumers, 35 countries, 2012

ILO: International Labour Organization.

Notes: Seven countries, written in bold typeface, voted to block the initiative to require prior informed consent for international shipment of chrysotile asbestos during the 2013 Conference of Parties to the Rotterdam Convention.

Data sources: Rotterdam Convention on the Prior Informed Consent Procedure for Certain Hazardous Chemicals and Pesticides in International Trade UNEP/FAO/TC/COP.6/20^4^ and Asbestos statistics and information.^5^

